# The urine output definition of acute kidney injury is too liberal

**DOI:** 10.1186/cc12784

**Published:** 2013-06-20

**Authors:** Azrina Md Ralib, John W Pickering, Geoffrey M Shaw, Zoltán H Endre

**Affiliations:** 1 Christchurch Kidney Research Group, Department of Medicine, University of Otago Christchurch, PO Box 4345, Christchurch 8140, New Zealand; 2 Intensive Care Unit, Christchurch Hospital, Private Bag 4710, Christchurch 8011, New Zealand; 3 Prince of Wales Hospital and Clinical School, University of New South Wales, High Street, Randwick, Sydney NSW 2031, Australia

## Abstract

**Introduction:**

The urine output criterion of 0.5 ml/kg/hour for 6 hours for acute kidney injury (AKI) has not been prospectively validated. Urine output criteria for AKI (AKI_UO_) as predictors of in-hospital mortality or dialysis need were compared.

**Methods:**

All admissions to a general ICU were prospectively screened for 12 months and hourly urine output analysed in collection intervals between 1 and 12 hours. Prediction of the composite of mortality or dialysis by urine output was analysed in increments of 0.1 ml/kg/hour from 0.1 to 1 ml/kg/hour and the optimal threshold for each collection interval determined. AKI_Cr _was defined as an increase in plasma creatinine ≥26.5 μmol/l within 48 hours or ≥50% from baseline.

**Results:**

Of 725 admissions, 72% had either AKI_Cr _or AKI_UO _or both. AKI_UO _(33.7%) alone was more frequent than AKI_Cr _(11.0%) alone (*P *<0.0001). A 6-hour urine output collection threshold of 0.3 ml/kg/hour was associated with a stepped increase in in-hospital mortality or dialysis (from 10% above to 30% less than 0.3 ml/kg/hour). Hazard ratios for in-hospital mortality and 1-year mortality were 2.25 (1.40 to 3.61) and 2.15 (1.47 to 3.15) respectively after adjustment for age, body weight, severity of illness, fluid balance, and vasopressor use. In contrast, after adjustment AKI_UO _was not associated with in-hospital mortality or 1-year mortality. The optimal urine output threshold was linearly related to duration of urine collection (*r*^2 ^= 0.93).

**Conclusions:**

A 6-hour urine output threshold of 0.3 ml/kg/hour best associated with mortality and dialysis, and was independently predictive of both hospital mortality and 1-year mortality. This suggests that the current AKI urine output definition is too liberally defined. Shorter urine collection intervals may be used to define AKI using lower urine output thresholds.

## Introduction

Urine output is a rapid bedside test for kidney function, and reduced output is the oldest known biomarker for acute kidney injury (AKI); historically described by Galen (200 CE) [1]. A rapid reduction of urine output may be the earliest indication of decreased kidney function. The Risk, Injury Failure, Loss, End stage (RIFLE) consensus definition of the Acute Dialysis Quality Initiative used urine output <0.5 ml/kg/hour for ≥6 hours to define AKI [2]. Subsequent AKI definitions have retained this criterion [3,4].

The urine output criterion (oliguric AKI) consistently classifies more patients as presenting AKI than the creatinine criteria [5-10]. Mortality is higher in those with oliguric AKI compared with those without AKI [10]. However, little is known about the comparative mortality for the creatinine criteria verse urine output criteria for AKI. Wlodzimirow and colleagues in a study of 260 patients note that the mortality rate for AKI diagnosed by creatinine alone was greater than for those diagnosed by either creatinine or urine output [8]. Cruz and colleagues noted that AKI defined by urine output was not an independent predictor of mortality [11]. Three studies have investigated the duration of oliguria in relation to creatinine or mortality. Oliguria (<0.5 ml/kg/hour) of any duration between 1 and 12 hours was only a fair predictor of subsequent development of AKI according to the RIFLE creatinine criterion [6], and mortality rates increased with increasing duration of oliguria [9,10]. To our knowledge, no study has attempted to define an optimum urine output threshold and duration of collection for AKI diagnosis.

We aimed to determine the ideal urine output threshold and collection duration by comparing various thresholds and durations with a predefined composite clinical outcome, namely death or need for dialysis.

## Materials and methods

Data were collected as part of the Fluid Loading in Acute Kidney Injury study, a prospective audit of fluid balance in the ICU of Christchurch Hospital. All patients admitted to the ICU from 1 October 2010 to 31 September 2011 were screened for inclusion. Patients were excluded if they were <17 years of age, stayed in the ICU for <24 hours, or were without recorded body weight, fluid input or urine output. Follow up was for 12 months. The Upper South A Regional ethics committee of New Zealand (URA/10/EXP/040) approved the study and waived the need for informed consent because only routinely available clinical information was collected.

Hourly fluid input and urine output data were extracted from ICU charts. The most recently documented body weight was used or, if unavailable, the weight reported by the patient or relatives or estimated from the patient demispan was used. Total body water (TBW) was calculated from age and weight according to the formulae [12]:

$$\text{Male}\;\text{total}\;\text{body}\;\text{water}=\text{2}0.0\text{3}-0.\text{1183}\times \text{age }\left(\text{years}\right)+0.\text{3626}\times \text{weight}\left(\text{kg}\right)$$

$$\text{Female}\;\text{total}\;\text{body}\;\text{water}=\text{14}.\text{46}+0.\text{2549}\times \text{weight}\left(\text{kg}\right)$$

For each patient, the total urine volume was recorded in moving blocks of duration 1, 2, 3, 4, 5, 7, 8, 9, 10, 11, and 12 hours. Each block began on entry to the ICU and was moved 1 hour at a time until the block ended 24 hours post entry. The minimum urine volume for each collection duration was recorded and divided by the body weight and duration of collection to yield 1-hour to 12-hour urine outputs in millilitres per kilogram per hour. The proportion of patients who died (in-hospital) or needed dialysis was calculated at urine output thresholds of 0.1 to 1 ml/kg/hour in steps of 0.1 ml/kg/hour for each collection duration.

Plasma creatinine was collected daily for 7 days and adjusted for cumulative fluid balance at the time of measurement as described by Macedo and colleagues [13]. Baseline creatinine was selected as: the pre-ICU creatinine within 7 to 365 days; or, if unavailable, the last post-discharge creatinine within 90 days; or, if unavailable, the on-admission creatinine. AKI based on plasma creatinine (AKI_Cr_) was defined as either an increase of >26.5 μmol/l within 48 hours or 50% within 7 days of admission [4]. AKI based on 6-hour urine output (AKI_UO_) was defined as urine output <0.5 ml/kg/hour using the lowest 6-hour period of urine output within the first 24 hours [4]. AKI severity stages were separately determined by change in creatinine or urine output according to the Kidney Disease: Improving Global Outcomes definition [4]. Severity of illness was assessed by the Acute Physiological and Chronic Health Evaluation (APACHE) II score [14], and the Simplified Acute Physiological Score II [15].

Results are presented as mean ± standard deviation for normally distributed variables or median (interquartile range) for non-normally distributed variables. All confidence intervals (CI) are 95%. For continuous variables, differences in two variables were analysed using an independent *t *test for normally distributed variables and the Mann-Whitney U test for non-normally distributed variables. For categorical variables, differences in proportions were analysed using the chi-square test.

Determination of whether the numbers of patients classified to each of AKI_Cr _and AKI_UO _were equal was determined by McNemar's test. The predictive performance of urine output for the combined outcome of in-hospital mortality or dialysis was assessed by the area under the curve (AUC) of the receiver operating characteristic (ROC) curve of the sensitivity over one minus specificity. The optimal threshold was defined as the biomarker concentration closest to the point on the ROC curve where sensitivity = 1 and specificity = 1.

The ideal urine output threshold for 6-hour collection for prediction of in-hospital mortality or dialysis need was investigated by determining: the ROC optimal threshold; the threshold with identical sensitivity to the creatinine-based AKI definition [16]; and the threshold that showed a marked increase in mortality or dialysis after being grouped in increments of 0.10 ml/kg/hour [17].

A multivariable logistic regression model for prediction of 30-day and 1-year mortality need was built with variables with *P *<0.1 under univariate analysis. Kaplan-Meier and Cox regression survival analyses were used for calculation of hazard ratios for 30-day and 1-year mortality for AKI_UO _and the 6-hour ideal urine output threshold.

The ROC optimal thresholds were determined from the ROC curves for 1-hour to 12-hour urine outputs for mortality or dialysis. A linear regression model was used to fit duration to optimal threshold. The relative risk for mortality or dialysis at each collection duration was calculated for patients with urine outputs below the regression line in comparison with those above the line.

Matlab 2011a (MathWorks, Natick, MA, USA), PRISM 6.0 (GraphPad, La Jolla, CA, USA) and SPSS version 19 (SPSS, Chicago, IL, USA) were used for statistical analyses.

## Results

There were 1,274 ICU admissions, of which 549 were excluded because admission was for <24 hours (*n *= 457), patients were aged <17 years (*n *= 45), there was missing fluid balance data (*n *= 8) or body weight was not recorded (*n *= 39). Of 725 ICU admissions, 522 (72%) had AKI (AKI_Cr _or AKI_UO_). The demographic profile and outcomes are shown in Table 1. Those patients with AKI were older, had higher body weight, had greater severity of illness (APACHE II score and Simplified Acute Physiological Score II), and were more likely to be hypotensive or need vasopressors. The mean APACHE II score was 18 ± 7, with 40% of patients (*n *= 287) with a score >20. Baseline plasma creatinine was greater in those with AKI, but there was no difference in the distribution of baseline creatinine selection criterion (*P *= 0.28). A higher incidence of AKI was observed in patients with cardiovascular diagnoses, kidney diagnoses or sepsis diagnoses as the primary diagnosis, and in post-surgical patients (*P *= 0.01). Hypertension, ischaemic heart disease, and diabetes mellitus were more common in those with AKI.

**Table 1:** Demographic profile, clinical characteristic and clinical outcome

Variable	Cohort (*n *= 725)	No AKI (*n *= 203)	AKI (*n *= 522)	*P *value
Age (years)	59 ± 18	53 ± 17	62 ± 17	<0.0001
Gender (male)	455 (62.7)	121 (59.6)	334 (63.9)	0.27
Weight (kg)	79 ± 19	74 ± 18	80 ± 19	<0.0001
Height (cm)	170 ± 10	170 ± 10	170 ± 11	0.87
APACHE II score	18 ± 7	16 ± 6	19 ± 7	<0.0001
SAPS II	40 ± 16	33 ± 14	43 ± 16	<0.0001
Vasopressor usage	366 (50.5)	84 (41.4)	282 (54.0)	0.002
Hypotension (MAP <60 mmHg)	384 (53.0)	97 (47.8)	287 (55.0)	0.08
Diuretic usage	28 (3.9)	5 (2.5)	23 (4.4)	0.22
Baseline plasma creatinine (µmol/l)	84 (71 to 102)	78 (69 to 89)	88 (72 to 107)	<0.0001
Baseline creatinine selection criterion				0.28
Pre-ICU 7 days to 1 year	466 (64.3)	122 (60.1)	344 (65.9)	
Final 90-day follow up	195 (26.9)	59 (29.1)	136 (26.1)	
First ICU admission	64 (8.8)	22 (10.8)	42 (8.0)	
Primary diagnosis class				0.01
Cardiovascular	70 (9.7)	12 (5.9)	58 (11.1)	
Endocrine/metabolic	11 (1.5)	0 (0)	11 (2.1)	
Gastrointestinal/hepatobiliary/pancreas	20 (2.8)	7 (3.4)	13 (2.5)	
Haematology/oncology/immunology	2 (0.3)	1 (0.5)	1 (0.2)	
Infective	3 (0.4)	2 (1.0)	1 (0.2)	
Renal	11 (1.5)	2 (1.0)	9 (1.7)	
Neurological	67 (9.2)	22 (10.8)	45 (8.6)	
Respiratory	145 (20.0)	48 (23.6)	97 (18.6)	
Trauma	64 (8.8)	24 (11.8)	40 (7.7)	
Postoperative surgical	259 (35.7)	61 (30.0)	198 (37.9)	
Cardiac surgery	152 (20.9)	30 (14.8)	122 (23.4)	
Other surgery	107 (14.8)	31 (15.3)	76 (14.6)	
Sepsis or septic shock	44 (6.1)	11 (5.4)	33 (6.3)	
Miscellaneous	29 (4.0)	13 (6.4)	16 (3.1)	
Baseline co-morbidities				
Hypertension	191 (26.3)	40 (19.7)	151 (28.9)	0.01
Cardiac failure	16 (2.2)	3 (1.5)	13 (2.5)	0.45
Ischemic heart disease	133 (18.3)	26 (12.8)	107 (20.5)	0.02
Chronic obstructive airways disease	54 (7.4)	12 (5.9)	42 (8.0)	0.33
Asthma	59 (8.1)	23 (11.3)	36 (6.9)	0.05
Diabetes mellitus	106 (14.6)	16 (7.9)	90 (17.2)	0.001
Kidney disease	56 (7.7)	11 (5.4)	45 (8.6)	0.15
Malignancy	62 (8.6)	15 (7.4)	47 (9.0)	0.49
Connective tissue/inflammatory disease	142 (19.6)	45 (22.2)	97 (18.6)	0.28
Dialysis in the ICU	41 (5.7)	0 (0)	41 (7.9)	<0.0001
Hospital mortality	100 (13.8)	19 (9.4)	81 (15.5)	0.03
Mortality at 1 year	151 (20.8)	30 (14.8)	121 (23.2)	0.01
Mechanical ventilation	562 (77.5)	159 (78.3)	449 (86.0)	0.01
Length of MV (hours)	23 (5 to 83)	18 (5 to 53)	25 (5 to 92)	0.02
Length of ICU stay (hours)	31 (21 to 72)	45 (31 to 95)	62 (39 to 148)	0.001
Length of hospital stay (days)	11 (6 to 20)	12 (6 to 22)	14 (8 to 25)	0.007

Data expressed as mean ± standard deviation, *n *(%), or median (lower quartile to upper quartile). AKI, acute kidney injury (by the creatinine definition or the urine output definition); APACHE, Acute Physiological and Chronic Health Evaluation II Score; MAP, mean arterial pressure; SAPS, Simplified Acute Physiology Score.

### Determination of the urine output threshold for 6-hour collection

#### Comparison of AKI_Cr _and AKI_UO_

One hundred and ninety-eight patients (27.3%) were diagnosed as both AKI_UO _and AKI_Cr _(Table 2). An additional 33.7% (*n *= 242) were diagnosed as AKI_UO _only and 11% (*n *= 80) as AKI_Cr _only (*P *<0.0001).

**Table 2:** Cross tabulation of AKI_UO _and AKI_Cr_

	AKI_Cr_		
	
AKI_UO_	No-AKI_Cr_	AKI_Cr_	Totals
No-AKI_UO_	203 (28.0)	80 (11.0)	283 (39)
AKI_UO_	244 (33.7)	198 (27.3)	442 (61)
Totals	447 (62)	278 (38)	725 (100)

Data expressed as *n *(%). McNemar *P *<0.0001. AKI_Cr_, acute kidney injury by the creatinine definition; AKI_UO_, acute kidney injury by the urine output definition.

One hundred patients died in hospital (13.8%), and 41 (7.9%) needed dialysis. Fourteen patients died after dialysis. There were therefore 127 patients with the composite outcome of in-hospital mortality or dialysis. Patients with both AKI_UO _and AKI_Cr _were 3.62 times more likely to die or need dialysis compared with those without AKI (Table 3). Patients with either AKI_UO _or AKI_Cr _were respectively 1.36 or 1.34 times more likely to die or need dialysis compared with those without AKI.

**Table 3:** Mortality or dialysis in AKI_UO _and AKI_Cr_

	Mortality or dialysis, *n *(%)^a^			Relative risk^b ^(95% CI)	
	
	No-AKI_Cr_	AKI_Cr_	Totals	No-AKI_Cr_	AKI_Cr_
No-AKI_UO_	19 (9.4)	10 (12.5)	29	1 (referent)	1.34 (1.20 to 1.39)
AKI_UO_	31 (12.8)	67 (33.5)	98	1.36 (1.20 to 1.40)	3.62 (3.21 to 3.94)
Totals	50	77	127		

^a^Number (percentage) of mortality or dialysis in each group. ^b^Relative risk of mortality or dialysis to the referent group of No-AKI_UO _and No-AKI_Cr_. AKI_Cr_, acute kidney injury by the creatinine definition; AKI_UO_, acute kidney injury by the urine output definition; CI, confidence interval.

#### Ideal 6-hour urine output thresholds to predict mortality or dialysis

The median 6-hour urine output was 0.42 (0.27 to 0.64) ml/kg/hour (minimum 0, maximum 4.05 ml/kg/hour). For a 6-hour collection interval, the AUC for prediction of mortality or dialysis was 0.70 (0.65 to 0.74), with an ROC optimal threshold of 0.31 ml/kg/hour.

The sensitivity of AKI_Cr _for prediction of mortality or dialysis was 61% (Table 4). The comparative threshold was defined as the 6-hour urine output that had the same sensitivity as AKI_Cr _for the same outcome. This value was also 0.31 ml/kg/hour. The specificity of urine output at this threshold (77%) was greater than for AKI_Cr _(66%).

**Table 4:** Prediction of mortality or dialysis

	Dialysis-free survival	Mortality or dialysis	Total	
AKI_Cr_				
No-AKI_Cr_	397	50	447	NPV = 89%
AKI_Cr_	201	77	278	PPV = 28%
Total	598	127	725	
	Sp = 66%	Sn = 61%		
Urine output				
≥0.31 ml/kg/hour	462	50	512	NPV = 90%
<0.31 ml/kg/hour	136	77	213	PPV = 34%
Total	598	127	725	
	Sp = 77%	Sn = 61%		

AKI_Cr_, acute kidney injury by the creatinine definition; NPV, negative predictive value; PPV, positive predictive value; Sn, sensitivity; Sp, specificity.

**Figure 1:**
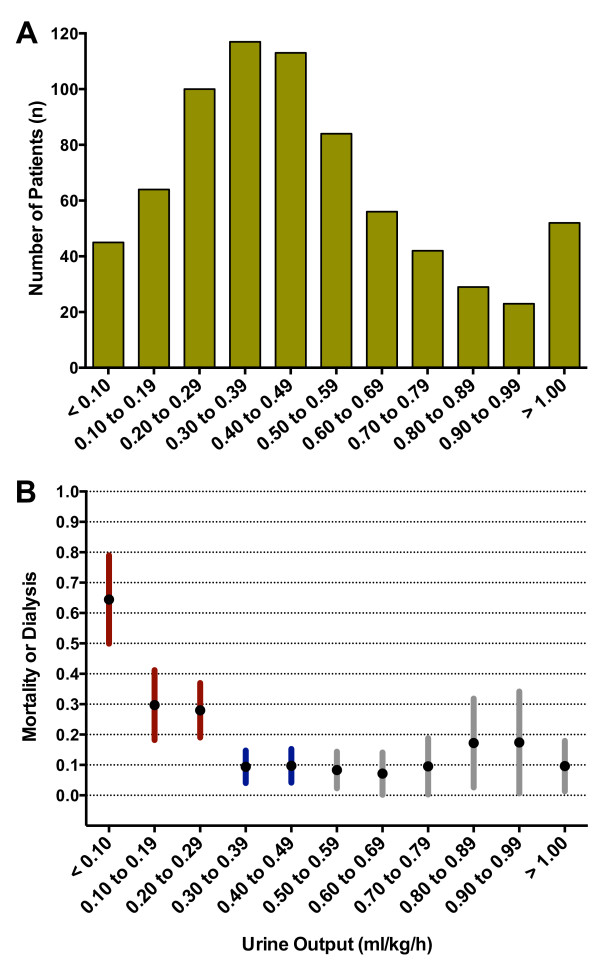
**Mortality or dialysis according to 6-hour urine output groups**. **(A) **Number in each group. **(B) **Proportion of mortality or dialysis in each group. Error bars represent 95% confidence intervals.

The distribution of patients in each of 11 intervals from <0.10 to >1.00 ml/kg/hour is shown in Figure 1A. There was a marked increase in mortality or dialysis need below 0.30 ml/kg/hour (Figure 1B). Above this threshold 10% (95% CI: 7 to 12%) died or needed dialysis compared with 36% (30 to 43%) below this threshold (*P *<0.0001). Of the 45 patients with urine output <0.1 ml/kg/hour, 29 (64%) died or needed dialysis.

#### Urine output <0.3 mg/kg/hour

Because each of the three methods for determining the 6-hour urine output threshold resulted in a threshold of approximately <0.3 mg/kg/hour (UO_<0.3_), we assessed its utility in comparison with AKI_UO _and AKI_Cr_.

Seventy-six (36%) UO_<0.3 _patients died or needed dialysis, compared with 51 (10%) for UO_≥0.3 _(*P *<0.0001). The sensitivity of UO_<0.3 _for prediction of mortality or dialysis was 61% (52 to 69%), specificity was 77% (73 to 80%), positive predictive value was 34% (29 to 41%), and negative predictive value was 90% (87 to 93%). Following adjustment for age, weight, APACHE II score, vasopressor use, fluid balance, presence of AKI_Cr _or dialysis, and baseline creatinine concentration (Table 5), the odds ratio for UO_<0.3 _was 3.31 (1.97 to 5.57) and that for AKI_UO _was 1.98 (1.14 to 3.43).

**Table 5:** Univariate analysis for prediction of mortality or dialysis

Variable	Odds ratio (95% CI)	*P *value
Age (years)	1.02 (1.01 to 1.03)	0.001
Body weight (kg)	0.98 (0.97 to 0.99)	0.005
APACHE II score	1.18 (1.14 to 1.22)	<0.0001
SAPS II	1.08 (1.07 to 1.10)	<0.0001
AKI_Cr _(yes or no)	3.00 (2.02 to 4.44)	<0.0001
Vasopressor use (yes or no)	1.65 (1.11 to 2.43)	0.01
Hypotension, MAP <60 mmHg (yes or no)	1.25 (0.85 to 1.84)	0.26
Diuretic use (yes or no)	1.60 (0.67 to 3.86)	0.29
Fluid balance (percentage/body weight)	1.16 (1.11 to 1.22)	<0.0001
Baseline creatinine (μmol/l)	1.005 (1.002 to 1.007	<0.0001
Urine output <0.5 ml/kg/hour (AKI_UO_)	2.50 (1.60 to 3.89)	<0.0001
Urine output <0.3 ml/kg/hour (UO_<0.3_)	5.21 (3.48 to 7.80)	<0.0001

AKI_Cr_, acute kidney injury by the creatinine definition; AKI_UO_, acute kidney injury by the urine output definition; MAP, mean arterial pressure; SAPS, Simplified Acute Physiological Score; UO_<0.3_, urine output <0.3 ml/kg/hour averaged over 6 hours.

As shown in Table 6, patients with both UO_<0.3 _and AKI_Cr _were 5.96 (5.05 to 6.92) times more likely to die or need dialysis compared with those with UO_≥0.3 _and No-AKI_Cr _(referent group). Patients with No-AKI_Cr _but with UO_<0.3 _were 3.29 (2.77 to 3.63) times and patients with AKI_Cr _and UO_≥0.3 _were 1.96 (1.76 to 2.05) times more likely to die or need dialysis, than those with UO_≥0.3 _and No-AKI_Cr _. The sensitivity of combining AKI_Cr _with UO_<0.3 _for death or dialysis was 79% and specificity was 55%, compared with combining AKI_Cr _with AKI_UO _where sensitivity was 85% and specificity was 31%.

**Table 6:** Mortality or dialysis in AKI_Cr _and urine output at a threshold of 0

Urine output	Mortality or dialysis, *n *(%)^a^		Relative risk^b ^(95% CI)	
	
	No-AKI_Cr_	AKI_Cr_	No-AKI_Cr_	AKI_Cr_
≥0.30 ml/kg/hour (UO_≥0.3_)	355	161		
Mortality or dialysis	27 (7.6)	24 (14.9)	1 (referent)	1.96 (1.76 to 2.05)
<0.30 ml/kg/hour (UO_<0.3_)	92	117		
Mortality or dialysis	23 (25.0)	53 (45.2)	3.29 (2.77 to 3.63)	5.96 (5.05 to 6.92)

AKI_Cr_, acute kidney injury by the creatinine definition; CI, confidence interval; UO_≥0.3_, urine output ≥0.3 ml/kg/hour averaged over 6 hours; UO_<0.3_, urine output <0.3 ml/kg/hour averaged over 6 hours. ^a^Number (percentage) of mortality or dialysis in each group. ^b^Relative risk of mortality or dialysis to the referent group of No-AKI_Cr _and UO_≥0.3_

**Figure 2:**
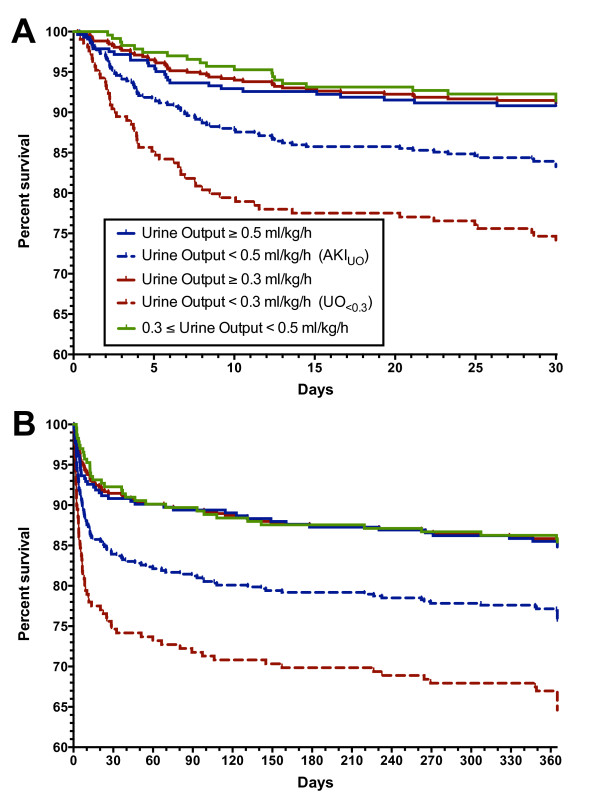
**Kaplan-Meier survival curves**. **(A) **In-hospital mortality. **(B) **One-year mortality. Log-rank test (Mantel-Cox): *P *<0.0001 for urine output <0.3 ml/kg/hour (UO_<0.3_) compared with urine output ≥0.3 ml/kg/hour in both cases (red lines), *P *<0.01 for urine output <0.5 ml/kg/hour (AKI_UO_) compared with urine output ≥0.5 ml/kg/hour in both cases (blue lines). There was no difference between urine output >0.5 ml/mg/hour (blue solid line) and 0.3 ≤urine output <0.5 ml/kg/hour (green line). (A) *P *=0.77, (B) *P *= 0.82.

Survival analysis (mortality only) comparing UO_<0.3 _with UO_≥0.3 _showed greater in-hospital mortality and 1-year mortality for UO_<0.3 _(*P *<0.0001; Figure 2). Similarly, AKI_UO _patients had greater in-hospital mortality (*P *= 0.0044) and 1-year mortality (*P *= 0.0027) than non-AKI_UO _patients (Figure 2). A survival analysis between three groups - urine output <0.3 ml/kg/hour (UO_<0.3_), 0.3 ≤urine output <0.5 ml/kg/hour, and urine output ≥0.5 ml/kg/hour (that is, not AKI_UO_) - showed no difference between 0.3 ≤urine output <0.5 ml/kg/hour and urine output ≥0.5 ml/kg/hour (*P *= 0.77; Figure 2). UO_<0.3 _patients were more than twice as likely to die in-hospital (hazard ratio: 2.25 (1.39 to 3.64)) or within 1 year (2.09 (1.42 to 3.08)) than UO_≥0.3 _patients after adjusting for covariates (age, weight, APACHE II score, vasopressor use, fluid balance, presence of AKI_Cr _or dialysis, and baseline creatinine). However, AKI_UO _patients were no more likely to die in-hospital (hazard ratio: 1.48 (0.89 to 2.45)) or within 1 year (1.43 (0.96 to 2.13)) than non-AKI_UO _patients.

### Duration of urine output

**Figure 3:**
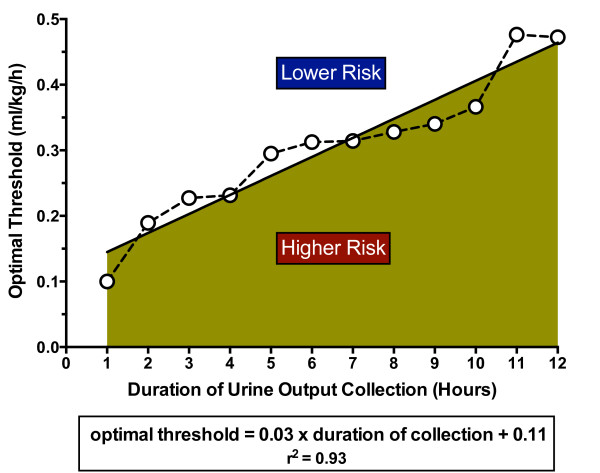
**Urine output threshold as a function of duration of urine collection**. Circles, actual optimal threshold at each duration; straight line, linear regression line (*r*^2 ^= 0.93).

For each incremental collection period between 1 and 12 hours we calculated the AUC for prediction of mortality or dialysis and compared it with the AUC for the 6-hour interval (Table 7). The optimal threshold for each collection interval was determined from the ROC curves and the relative risk of mortality or dialysis for those below the threshold compared with those above was calculated. The AUC of urine output assessed over periods of 3 to 5 hours and 7 to 12 hours were not different from the 6-hour period (*P *>0.1; Table 7). The ROC optimal threshold for predicting mortality or dialysis was linearly correlated with duration of urine output (*r*^2 ^= 0.93; Figure 3). The regression line for the optimal threshold was calculated as follows:

**Table 7:** Areas under the curve for varying durations of urine output from 1 to 6 hours

Duration of urine output (hours)	AUC (95% CI)	*P *value^a^	Optimal threshold (ml/kg/hour)	Relative risk (95%CI)
12	0.68 (0.63 to 0.73)	0.054	0.47	2.69 (1.93 to 3.75)
11	0.68 (0.64 to 0.73)	0.095	0.48	2.53 (1.83 to 3.50)
10	0.69 (0.64 to 0.73)	0.087	0.37	2.52 (1.83 to 3.47)
9	0.69 (0.64 to 0.74)	0.2	0.34	2.94 (2.14 to 4.06)
8	0.70 (0.65 to 0.74)	0.64	0.33	3.27 (2.38 to 4.49)
7	0.70 (0.65 to 0.74)	0.76	0.31	3.39 (2.47 to 4.65)
6	0.70 (0.65 to 0.74)	1	0.31	3.68 (2.68 to 5.04)
5	0.69 (0.65 to 0.74)	0.23	0.30	3.45 (2.53 to 4.72)
4	0.71 (0.66 to 0.75)	0.47	0.23	3.61 (2.62 to 4.99)
3	0.69 (0.64 to 0.73)	0.17	0.23	3.03 (2.22 to 4.14)
2	0.66 (0.62 to 0.71)	0.036	0.19	2.68 (1.94 to 3.68)
1	0.61 (0.56 to 0.66)	0.0003	0.10	1.77 (1.27 to 2.48)

Areas under the curve (AUCs) of varying durations of urine output from 1 to 6 hours for prediction of mortality or dialysis and relative risk for those below the threshold compared with those above the threshold. Comparisons were made in reference to the AUC of average hourly urine output over 6-hour period with the DeLong method. CI, confidence interval. ^a ^*P *value for difference relative to 6-hour urine output.

$$\text{Optimal}\;\text{threshold}\left(\text{ml}/\text{mg}/\text{hour}\right)=0.0\text{3}\times \text{duration}\;\text{of}\;\text{urine}\;\text{collection}\left(\text{hours}\right)+0.\text{11}$$

Patients with urine output below the regression line were at greater risk of dying or needing dialysis, whereas those patients above the regression line were at lower risk. The relative risk of dying or needing dialysis below the line for a 6-hour urine output was 3.68 (2.68 to 5.04) (Table 7).

## Discussion

In this prospective study, a 6-hour urine output threshold of 0.3 ml/kg/hour was most clearly associated with the composite clinically important outcomes of mortality or dialysis, while urine output <0.5 ml/kg/hour was not associated with in-hospital mortality after adjustment for covariates. A urine output <0.3 ml/kg/hour predicted in-hospital mortality and 1-year mortality after adjusting for age, body weight, severity of illness, fluid balance, and vasopressor use. A 6-hour urine output between 0.3 and 0.5 ml/kg/hour was not associated with increased mortality compared with urine output >0.5 ml/kg/hour.

AKI was first defined by consensus in 2003 [2]. Alongside a change in the glomerular filtration rate (GFR) criterion that could be measured by its surrogate, a change in plasma creatinine concentration [2,18], AKI could be defined by a decrease in urine output to <0.5 ml/kg/hour for 6 hours. The duration and urine output volume were defined by consensus not by physiology. The urine output criterion was incorporated without alteration in subsequent AKI definitions [3,4].

Urine output has three advantages over plasma creatinine. Firstly, low urine output is categorically defined and is not reliant on knowing a baseline urine output, in contrast to plasma creatinine definitions that depend on a baseline creatinine, which is often unknown and therefore must be estimated, a process that introduces significant errors [19-21]. Secondly, a reduced urine output is potentially the first indication of kidney dysfunction especially in critical care settings where hourly urine outputs are routinely measured. Thirdly, recent evidence suggests that in some circumstances creatinine production is dramatically reduced, rendering its use inaccurate as a surrogate for GFR [22]. Urine output changes were shown to precede changes in plasma creatinine and to follow closely the pattern of GFR changes [23]. However, the urine output criterion has not been well validated compared with that of the plasma creatinine criterion. The method of assessing oliguria was not specified. The guideline did not specify whether the reduction in urine output should be defined by the average flow over 6 hours, or from a persistent reduction over the 6 consecutive hours [4]. Using the plasma creatinine Acute Kidney Injury Network definition as the reference standard, Macedo and colleagues compared three different methods of urine output assessment: persistent reduction every hour, average reduction over a moving 6-hourly interval, and a 6-hour interval matching the nursing shift in 317 critically ill patients [9]. Averaging urine output over 6 hours was more sensitive than six successive hourly urine output measurements. We used averaging in this analysis.

At a threshold of 0.5 ml/kg/hour there was a higher incidence of AKI utilising the urine output definition compared with the plasma creatinine-based definition (61% vs. 38%). Thirty-four per cent of patients with AKI based on urine output were not diagnosed AKI by the plasma creatinine changes. Previous studies have also reported a higher incidence of AKI by urine output compared with the creatinine criteria [5-10]. Eleven per cent (*n *= 80) of our patients had AKI based on creatinine, but not urine output, and they may be described as nonoliguric AKI [10]. These patients may also be those with recovering AKI in that creatinine had yet to fall to baseline although GFR had already recovered [24].

A urine output below an average of 0.3 ml/kg/hour over 6 hours was associated with higher mortality and dialysis need than 0.5 mg/kg/hour over 6 hours. Both the ROC optimal threshold for prediction of mortality or dialysis and the threshold that had the same sensitivity as AKI_Cr _for predicting mortality or dialysis were, serendipitously, 0.3 ml/kg/hour over 6 hours. Interestingly, acute oliguria is classically defined as urine output <400 ml/day, which is equal to 0.24 ml/kg/hour in a 70 kg human [25,26]. This is the minimum urine required to eliminate 300 mOsm/day in a maximum urine concentration of 1,200 mOsm/kg. Defining AKI as daily urine output <500 ml or creatinine >3.5 mg/dl, Teixerira and colleagues showed that the mean 24-hour urine volume was lower in those who died compared with survivors [27].

Urine output is influenced by many factors including fluid balance, presence of hypotension, and the use of diuretics or vasopressors. These factors were included in the analysis of prediction of hospital mortality and 1-year mortality. After adjusting for these covariates, urine output <0.3 ml/kg/hour, but not <0.5 mg/kg/hour, was predictive of hospital mortality and 1-year mortality. These patients were approximately twice as likely to die compared with those with urine output >0.3 ml/kg/hour.

If a 6-hour urine output threshold of 0.3 ml/kg/hour was to be used to define AKI rather than 0.5 ml/kg/hour, then the overall AKI incidence (defined by urine output or AKI_cr_) will fall. In this study it would have decreased from 72% (522/725) to 51% (370/725). Of the 370 patients, 92 would have been diagnosed AKI by the modified urine output criterion alone. These have a meaningful increase in mortality or dialysis risk (Table 6) and should not be overlooked. A less liberal urine output criterion necessarily decreased the sensitivity of the combined urine output and creatinine AKI definition for death or dialysis by 6%, which was compensated by an increase in specificity of 24%.

For clinical applicability and earlier detection of AKI, it may be feasible to assess urine output over <6 hours. Reduction of urine output over 2 consecutive hours has been suggested [28]. Prowle and colleagues investigated different durations of consecutive hours of oliguria from 1 to 12 hours in a multicenter study involving 239 patients. Urine output of 4 hours or more best discriminated between AKI_Cr _and No-AKI_Cr _[6]. Recently Mandelbaum and colleagues retrospectively interrogated a database of over 25,000 ICU patients and observed an association between urine output thresholds and duration with mortality, and with renal replacement therapy [29]. Urine outputs >0.3 ml/kg/hour resulted in little or no increase in mortality until durations exceeded the 12 hours we assessed in this study. Even less sensitivity of urine output was found for renal replacement therapy. We found a linear relationship between the ROC optimal threshold and duration of urine output for discriminating between mortality or dialysis need and dialysis free-survival from to 0.14 ml/kg/hour for 1 hour to 0.47 ml/kg/hour for 12 hours. There was no difference in relative risk for this outcome as a function of duration from 3 to 9 hours. Although a shorter duration of urine output assessment may provide earlier diagnosis, this may be more susceptible to extraneous factors. A longer period of assessment >9 hours is less sensitive and may miss acute changes.

Urine output provides rapid assessment of kidney function, and is often used to guide fluid resuscitation in the critical care setting. A higher target of urine output tends to lead to a higher fluid balance, especially in the setting of nonvolume responsive kidney function. Because there is increasing evidence of a detrimental effect of cumulative fluid balance [30-32], a lower minimum urine output target may reduce fluid accumulation and its associated adverse outcomes. This is akin to permissive hypercapnia in ventilator-induced lung injury, where understanding the adverse effect of high tidal volume ventilation has resulted in a shift in clinical paradigm to accept a higher level of hypercapnia [33]. Similarly, a shift amongst clinicians to accept a lower limit of urine output may reduce the adverse effects associated with fluid accumulation. Nevertheless, the occurrence of oliguria should not be considered in isolation. Other clinical predictors of AKI may need to be considered, including plasma creatinine changes, haemodynamic stability, vasopressor usage, and injury biomarkers [6]. To establish the biological plausibility of using low urine output as a marker of AKI, a study investigating the biological and temporal relationship of low urine output with other physiological endpoints including recovering verse nonrecovering plasma creatinine, peak plasma creatinine, urinalysis and kidney injury specific biomarkers is necessary.

### Study limitations

This study has several limitations. First, the study was performed in only a single centre, so the generalisability of this study is significantly limited by the small sample size and the limited case mix. We note, however, a retrospective study by Mandelbaum and colleagues in more than 25,000 ICU patients that observed urine output <0.3 ml/kg/hour for at least 5 hours was associated with increased mortality [29].

Second, this general ICU had a mean APACHE II score of 18 ± 7 (range 3 to 47). Forty per cent of patients (*n *= 287) had APACHE II score >20. While APACHE II scores were taken into account in multivariate analysis, because other ICUs may include higher proportions of more severely ill patients, we recommend that a similar analysis be carried out in such a cohort to ensure that there is no bias introduced because of illness severity.

Third, of the 1,274 total ICU admissions screened, approximately one-third were admitted for <24 hours and these patients were excluded from the analysis. These patients included both those who did not survive 24 hours and those who were discharged within 24 hours. This exclusion may bias the results if a significant portion of those discharged early had low urine output.

Fourth, body weight was determined indirectly from the most recent body weight documented in medical records, or as reported by a patient or relative. In 6% (46 cases) these data were not available and body weight was estimated from the patient demispan. This estimate may affect the interpretation of the result if the body weight used was systematically either over or under the patient's true body weight. However, an analysis of urine output without factoring in body weight showed that a threshold of minimum 6-hourly urine output of 20 ml/hour, which is the equivalent of 0.3 ml/kg/hour in an average 70-kg person, best associated with mortality.

Finally, diuretic administration may lead to misclassification of AKI_UO _or UO_<0.3_, and hence may mitigate the impact on these classifications in predicting outcome. A sensitivity analysis of patients without diuretic administration (*n *= 697) yielded similar odds ratios compared with the entire cohort (adjusted odds ratios for UO_<0.3 _of 3.89 (2.25 to 6.70) and AKI_UO _of 2.41 (1.35 to 4.31)).

## Conclusions

The threshold for 6-hour urine output of an average of 0.3 ml/kg/hour was best associated with mortality or dialysis. This threshold was independently predictive of hospital mortality and 1-year mortality after adjustment for covariates. This suggests that the current AKI urine output definition may be too liberally defined. The optimal threshold of urine output was linearly related to duration. We recommend that a simple bedside formula be applied to identify AKI; namely, for a duration of collection between 3 and 9 hours:

$$\text{Urine}\;\text{output}\left(\text{ml}/\text{kg}/\text{hour}\right)<0.0\text{3}\times \text{duration}\;\text{of}\;\text{collection}\left(\text{hours}\right)+0.\text{11}$$

## Key messages

• A urine output threshold for defining AKI of <0.5 ml/kg/hour over 6 hours is too liberal.

• A threshold of <0.3 ml/kg/hour over 6 hours better fits with the plasma creatinine definition of AKI by having a similar sensitivity for a clinically relevant outcome.

• AKI may be diagnosed from urine output in as little as 2 to 3 hours.

• The urine output threshold for AKI should be adjusted for duration of urine collection.

• AKI may be defined as: Urine output (ml/kg/hour) <0.03 × duration of collection (hours) + 0.11, where the duration is between 3 and 9 hours.

## Abbreviations

AKI: acute kidney injury; AKI_Cr_: acute kidney injury by the creatinine definition; AKI_UO_: acute kidney injury by the urine output definition; APACHE: Acute Physiological and Chronic Health Evaluation; AUC: area under the curve; GFR: glomerular filtration rate; ROC: receiver operating characteristic; UO_≥0.3_: urine output ≥0.3 ml/kg/hour averaged over 6 hours; UO_<0.3_: urine output <0.3 ml/kg/hour averaged over 6 hours.

## Competing interests

The authors declare that they have no competing interests.

## Authors' contributions

AMdR collected the data, performed the analysis, participated in data interpretation, wrote the first draft of the manuscript and approved the final manuscript. JWP oversaw the analysis, and participated in data interpretation, manuscript writing and approval. GMS assisted with data collection, participated in data interpretation and manuscript writing, and approved the manuscript. ZHE participated in data interpretation, manuscript writing and approval. All authors read and approved the final manuscript.
